# Prehospital Cold-Water Immersion for Undifferentiated Heat Stroke: A Case Series

**DOI:** 10.5811/cpcem.61684

**Published:** 2026-08-31

**Authors:** David Lindow, Bryan Everitt, Nick Smith, Michael Wells, Robert Dickson, Casey Patrick

**Affiliations:** * University of Houston, Department of Emergency Medicine, Kingwood, Texas; † Allegiance Mobile Health Emergency Medical Services, Corpus Christi, Texas; ‡ Montgomery County Hospital District Emergency Medical Services, Conroe, Texas; § Baylor College of Medicine, Department of Emergency Medicine, Houston, Texas

**Keywords:** heat stroke, emergency medical services, cold water immersion, resuscitation, critical care

## Abstract

**Introduction:**

Heat stroke is a time-sensitive condition that requires early intervention to prevent morbidity and mortality. Heat-related deaths are also increasing. Multiple studies have evaluated various cooling methods and assessed their effectiveness, safety, and practicality across diverse settings and populations. Earlier and faster cooling leads to improved patient outcomes. The National Association of Emergency Medical Services Physicians recommends initiating prehospital cold-water immersion before emergency medical services (EMS) transport.

**Case Series:**

This case series evaluated the feasibility and effectiveness of prehospital cold-water immersion initiated before and continued during EMS transportation from the scene to emergency department arrival in this critically ill patient population.

**Conclusion:**

Rapid initiation and maintenance of prehospital cold-water immersion in undifferentiated heat stroke was both feasible and effective. Over the course of the case series, no deaths were reported, and all patients were eventually discharged home.

## INTRODUCTION

Heat stroke is a severe condition that requires early intervention to prevent long-term sequelae, morbidity, and mortality.^1^–^4^ Heat stroke is classified into two distinct types: classical and exertional. Classical heat stroke typically affects individuals at the extremes of age, owing to a combination of environmental and underlying medical factors. In contrast, exertional heat stroke typically occurs in younger, healthier individuals. Heat stroke develops when the body loses its ability to regulate its core temperature. The elevated body temperatures during heat stroke have been shown to cause denaturing of proteins on a molecular level, leading to cellular death and organ failure, including the central nervous system, heart, kidney, lungs, liver, spleen, muscle, and intestines.^1^,^2^

Death due to heat-related illnesses, such as heat stroke, has been a prevalent problem in the United States. Between 2018 and 2020, the Centers for Disease Control and Prevention recorded 3,066 heat-related deaths, with the highest percentage occurring among individuals 55 to 74 years of age.^1^ One study reported that exertional heat-related deaths are the top noncardiac cause of death in laborers.^2^ Although it is difficult to get an accurate account of heat-related deaths due to multiple variables, heat-related illnesses and mortality increase significantly during heat waves and periods of elevated temperatures.^3^ The earlier a patient can be treated and cooled, the lower the risk of long-term sequelae and death.^3^–^6^ The most effective current treatment for heat-related illness is to rapidly cool the body’s core temperature early.^2^,^4^,^6^

Numerous studies have been conducted to evaluate various cooling methods, assessing their effectiveness, safety, and practicality in diverse settings and populations. These methods include passive cooling with rest, cold showers,^7^ warm cooling,^8^ cold water cooling,^9^ ice water cooling, ice sheets,^10^–^12^ tarp cooling,^13^–^16^ body bag use,^17^ cold fluid intravenous (IV) infusion,^11^ and ice packs. Most of the studies conclude that more rapid cooling improves outcomes and is associated with fewer long-term sequelae and negative neurological outcomes.^4^–^6^,^18^,^19^ Cold water immersion (CWI) is the most effective cooling method, decreasing core body temperature at a rate of 0.20 °C/min to 0.36 °C/min..^5^–^7^,^20^ Some studies challenge the general consensus, arguing that cooling too rapidly is dangerous, with potential risks including shock, dysrhythmias, hypotension, and seizures; suggesting that warmer temperatures should be used instead.^8^,^21^ Although the risks mentioned above can and often do occur, they are manageable if proper precautions are taken with medications and countermeasures in place.^6^ In an 18-year retrospective study conducted at the Falmouth Road Race, Falmouth, MA,. patients with exertional heat stroke treated with cold water immersion (CWI) in water less than 10 °C cooled at at an average rate of 0.22 °C/min.^22^ Although that study did not track incidence rates for those specific complications, the overall survival rate was 100%, and 93% of the 270 patients were discharged directly from the medical tent.^22^ Cold-water immersion has been shown to be safe and effective, such that it can be performed without serial rectal temperature measurements and can be based on initial temperature and immersion time.^22^,^23^

Cold-water immersion for heat stroke treatment has become the standard of care, with proposed algorithms to guide its successful implementation in emergency department (ED) and athletic event settings.^6^,^7^,^24^ Tishukaj noted that exertional heat stroke patients are 3.0 to 3.7 times less likely to die when they are treated with CWI prior to transport.^2^ The National Association of Emergency Medical Services Physicians examined these cases and others and considered the risks, benefits, and outcomes. Their recommendation is for CWI with complete immersion to the level of the clavicles in water at 10 °C or below.^25^ Along with this recommendation, they also suggest initiating cooling of the patient prior to transportation, which aligns with the study conducted by Tishukaj.^26^ Most studies have been conducted in young, healthy individuals, focusing on exertional heat stroke rather than classic heat stroke. However, there have been a few case reports in the literature regarding CWI treatment for classic heat stroke in elderly patients, which have proven safe and effective.^16^,^26^,^27^

Cold-water immersion initiation prior to transportation is the standard of care for treating heat stroke.^4^–^6^ One case series featuring five elderly patients with classical heat stroke reported treatment on scene with CWI, using water with continuous flow from a fire hydrant prior to transportation.^26^ While effective cooling prior to emergency medical services (EMS) transport was shown, in one instance it took 69 minutes to decrease the core temperature to the point they could safely remove the patient from the water and transport them to the hospital.^27^ This highlights the need to balance time to patient cooling and the necessity of rapid hospital transport for additional diagnostic and treatment efforts. Emergency medical services must have the equipment and capabilities to quickly initiate CWI on scene, package the patient, and continue cooling during transport. However, due to limited space, limited access to reliable ice/water, and motion during transport, prehospital CWI presents unique challenges, raising questions about its prehospital feasibility and effectiveness.

*CPC-EM Capsule*

What do we already know about this clinical entity?

*Heatstroke is a time-sensitive emergency. Rapid cooling and emergency department transport are the foundations of prehospital care.*

What makes this presentation of disease reportable?

*Limited evidence exists to support initiating cold-water immersion for undifferentiated heatstroke in the civilian prehospital setting.*

What is the major learning point?

*Prehospital cold-water immersion for critically ill heatstroke patients is feasible and effective in a ground-based emergency medical services system.*

How might this improve emergency medicine practice?

*These preliminary data should support larger prospective studies of cold-water immersion for the treatment of prehospital heatstroke.*

This case series summarizes nine patients treated under a previously approved and implemented EMS protocol for initiation of CWI in patients with undifferentiated prehospital heat stroke. The participating agency is a ground-based EMS service in the greater Houston area that employs nearly 350 EMS clinicians, including paramedics, advanced emergency medical technicians, and emergency medical technicians and services all emergency dispatch response and transport for an area of approximately 1,100 square miles. This area has a population of roughly 750,000, aimed at which the service receives approximately 90,000 service calls per year. The nine patients in this case series were treated between July 18, 2023, and September 14, 2024.

This case series was part of the continuous quality improvement of the CWI protocol, which was implemented on May 25, 2022 (Figure). To ensure patient safety, all CWI cases were thoroughly reviewed by EMS medical direction for protocol compliance and clinical decision-making. Direct paramedic feedback was provided when necessary and appropriate. Hospital data was obtained as part of the continuous quality improvement project, as well.

Before protocol rollout, all EMS clinicians attended a mandatory one-hour lecture-based protocol and heat stroke education session, followed by a training video. The CWI protocol inclusion criteria include suspected heat stroke-related illness, defined in the protocol as altered mental status accompanied by a core temperature of greater than 40.6 °C (105 °F). Advanced age defined as older than 60 years, was considered a relative contraindication due to the increased risk of infectious causes of fever, and protocol teaching emphasized the necessity of obtaining a thorough history prior to initiation of CWI. Online consultation with permission from either a district chief/supervisor paramedic, or medical direction was required prior to initiation of CWI. Equipment required for the procedure included an immersion bag, ice, and water. Patients were undressed and placed in the bag on the stretcher with the head elevated to a minimum of 15°. The bag was then filled with ice and water to the patient’s chest (Image). Emergency medical services (Table 1) and ED/hospital data (Table 2) were collected.

## CASE SERIES

### Case #1

A 67-year-old man was initially found unconscious by his family on his bed. The family reported that the air conditioner in the house had not been functioning properly, and the interior of the house had become extremely hot. Emergency medical services found the patient unresponsive with rapid, shallow breathing, tachycardia, and a reported Glasgow Coma Scale (GCS) score of 3. The patient’s airway was addressed with bag-valve mask ventilations. The rectal temperature was 41.8 °C (107.3 °F). The CWI protocol was initiated, bilateral IV access sites were established, and a cold 0.9% normal saline bolus was administered. The patient was intubated with ketamine and rocuronium due to concerns for a lack of adequate airway protection.

Approximately 20 minutes after immersion, the patient’s blood pressure decreased to 89/57 mm Hg. He received a 20 mcg push dose of IV epinephrine, which resulted in an appropriate increase to 119/82 mm Hg. Eight minutes later, he received a second dose, which increased the blood pressure to 149/75 mm Hg. Just before ED arrival, a repeat rectal temperature was recorded as 39.6 °C (103.3 °F). Hospital data demonstrated an elevated initial lactic acid of 3 mmol/L, (reference range, < 2 mmol/L), with normal aspartate aminotransferase (AST) and alanine aminotransferase (ALT) (AST 5-40 U/L and ALT 7-56 U/L) values. The patient was admitted to the intensive care unit (ICU) with a length of stay (LOS) of seven days and 16 hours before being discharged home.

### Case #2

A 72-year-old male was found unresponsive in direct sunlight by his family. Emergency medical services described the patient to be unresponsive with agonal, ineffective breathing and hot to the touch, with GCS 4. Emergency medical services provided airway suctioning and immediately administered oxygen via bag-valve mask. The patient’s rectal temperature was recorded as 41.3 °C (106.4 °F). External cooling attempts were initiated by placing ice packs in the armpits, groin, and behind the neck. An IV was established, and the patient received a 0.9% normal saline bolus and the initiation of the CWI. Ten minutes after cooling initiation and six minutes after CWI initiation, the patient was noted to be hypotensive at 82/52 mm Hg. The patient was administered a 20-mcg push dose of epinephrine, which increased blood pressure to 127/64 mm Hg. The patient’s GCS score at this time also increased to 7. The respiratory status was still inadequate, requiring bag-valve mask ventilations; so, the patient was intubated using ketamine and rocuronium. Approximately 12 minutes after the initial dose of epinephrine, the blood pressure decreased to 81/44 mm Hg, and a second 20 mcg push-dose of epinephrine was administered. The patient again responded appropriately, with blood pressure increasing to 109/69 mm Hg. Upon ED arrival, the patient’s initial lactic acid was elevated at 5.9 mmol/L, and AST/ALT were elevated to 56 U/L and 258 U/L, respectively. The patient also had acute kidney injury (AKI) with an initial creatinine of 8.8 mg/dL (reference range, 0.7–1.3 mg/dL) with associated hypokalemia of 2.2 mEq/L (3.5–5.0 mEq/L). While at the hospital, the patient was admitted to the ICU and stayed for nine days, 18 hours before being discharged home.

### Case #3

A 78-year-old man was initially found unconscious in his vehicle by bystanders who removed him and called emergency dispatch. Upon EMS arrival, the patient had a GCS score of 3. The prehospital electrocardiogram was noted to be sinus tachycardia, and the patient was tachypneic and hypoxic. Emergency medical services provided supplemental oxygen via bag-valve mask at 15 liters/min. He was found to have a rectal temperature of 40.9 °C (105.6 °F). Cooling measures were started by placing ice packs in the patient’s armpits, groin, and neck. Intravenous placement was initiated, a cold 0.9% normal saline bolus was administered, and CWI was initiated. Upon ED arrival, rectal temperature was reassessed and noted to have decreased to 39.3 °C (102.7 °F). The patient was reported to be more alert and answered some questions appropriately with a documented GCS score of 13. The time from CWI initiation to the repeat rectal temperature was 22 minutes. Lactic acid and AST/ALT were normal. The patient did have AKI with an initial creatinine of 4.3 mg/dL. He was admitted to the hospital floor and remained for three days, 20 hours before discharge home.

### Case #4

A 60-year-old woman was found unresponsive in her home. Upon EMS arrival, the patient had an initial GCS score of 3 but then began seizing with oxygen saturations of 60%. Paramedics immediately began addressing the hypoxia by using a bag-valve mask at 15 L/min and placing a nasopharyngeal airway. She was given 10 mg intramuscular midazolam for the seizure, which subsequently ceased. The patient’s rectal temperature was found to be 40.9 °C (105.6 °F). Cold-water immersion was initiated, an IV was established, and the patient received a cold 0.9% normal saline fluid bolus. Two minutes after CWI initiation, the blood pressure dropped to 81/39 mm Hg. A 20-mcg push-dose of epinephrine was administered, resulting in an appropriate response, with blood pressure increasing to 133/69 mm Hg. During transport, the patient was observed opening her eyes and withdrawing from painful stimuli. Upon ED arrival, 17 minutes after CWI initiation, the patient’s rectal temperature was 39.7 °C (103.5 °F). The patient’s mentation also notably improved, as evidenced by a GCS score of 9. The patient’s initial lactic acid was elevated at 2.5 mmol/L, and AST/ALT were normal. The patient had mild AKI with an initial creatinine of 1.9 mg/dL. The patient was admitted to the ICU and stayed for five days before being discharged home.

### Case #5

A 49-year-old man was found altered by coworkers while working outside. Upon EMS arrival, the patient was reported to be altered, listless, pale, and hot to the touch. The patient had a GCS score of 7, with a rectal temperature of 42.7 °C (108.9 °F). He was placed on oxygen at 15 L/min. via a nonrebreather mask. Cold packs were applied to his groin and armpits. An IV was started, and a cold 0.9% normal saline bolus was infused. The patient became combative, requiring sedation using 100 mg IV ketamine. Cold-water immersion was partially initiated; however, the crew had no ice available. During transport, EMS stopped at a gas station and purchased ice to cool the patient further. The patient again became more combative and was given a second dose of 100 mg IV ketamine. The patient required suctioning to control secretions but maintained intact airway reflexes. Just prior to arrival at the hospital, seizure activity was observed, and 5 mg IV midazolam was administered. Upon ED arrival, 20 minutes after initiating CWI, the patient’s rectal temperature had decreased to 40 °C (104.0 °F). Lactic acid was normal, but AST/ALT were elevated to 339 and 245 U/L, respectively. The patient had mild AKI with an initial creatinine of 1.8 mg/dL and rhabdomyolysis with creatine kinase of 10,750 IU/L (reference range, 25–320 U/L). The patient was intubated in the ED, admitted to the ICU, and stayed for seven days and five hours before being discharged home.

### Case #6

A 51-year-old man was found outside with altered mentation. Upon EMS arrival, the patient was altered, tachycardic, tachypneic, hot to the touch, with a GCS score of 10. The patient’s initial oral temperature was recorded as 40.6 °C (105.0 °F). He was placed on oxygen at 15 L/min due to oxygen saturations of 91%, and cold packs were applied to his neck, axillae, and groin. Cold-water immersion was initiated, intraosseous (IO) access was established, and a cold 0.9% normal saline fluid bolus was infused. Two mg IO midazolam was administered for suspected focal seizure activity. An IV was then established, and continued fluid administration was maintained. Upon ED arrival, 11 minutes after initiation of CWI, the patient’s temperature was 39.4 °C (102.9 °F). The patient’s initial lactic acid, AST/ALT, and creatinine were normal. The patient was admitted to the ICU and had a hospital LOS of 17 days and 19 hours before being discharged home.

### Case #7

A 60-year-old man was found outside with altered mental status. Upon EMS arrival, the patient was found with a GCS score of 10. The patient had minor abrasions but otherwise had no obvious signs of significant injury. The initial rectal temperature was 40.7 °C (105.3 °F), prompting initiation of CWI. An IV was established, and the patient received a cold 0.9% normal saline bolus. On ED arrival, the patient was noted to be speaking, answering questions, and following commands with a GCS score of 14. Time from EMS arrival to CWI initiation was 29 minutes, and 36 minutes from CWI initiation to hospital arrival. The patient’s initial temperature was 37.3 °C (99.1 °F). His initial lactic acid, AST/ALT, and creatinine were normal. He was admitted to the hospital floor and LOS was 19 hours before being discharged home.

### Case #8

A 70-year-old man was found with unexplained confusion, and the family called emergency dispatch. Upon EMS arrival, the patient was found altered with a GCS score of 12. Vital signs revealed hypotension (73/48 mm Hg), tachycardia (127 beats/min[bpm]), and a rectal temperature of 41.3°C (106.4 °F). Oxygen was administered with a nasal cannula at six L/min. Additionally, an IV was started, a 20-mcg push dose of epinephrine was administered, a cold 0.9% normal saline bolus was initiated, and CWI was performed. During transport, the patient’s mental status improved, and he answered questions appropriately. The mentation improved to a GCS score of 15. There were five minutes from EMS arriving on scene to CWI initiation and 40 minutes from CWI initiation to hospital arrival; the initial ED temperature was 36.4 °C (97.6 °F). The patient’s initial lactic acid was elevated at 5.9 mmol/L, and AST/ALT were normal. The patient had an AKI with an initial creatinine of 3.9 mg/dL. He was admitted to the ICU and LOS was 14 days and 20 hours before being discharged home.

### Case #9

A 50-year-old woman was found with severe altered mentation. EMS noted initial hypotension (66/47 mm Hg) and tachycardia (122 bpm), with a GCS score of 6. Initial rectal temperature was 42.1 °C (107.8 °F). A nonrebreather oxygen mask was applied, and cold packs were placed in the groin, armpits, and the back of the neck. An IV was established, a cold 0.9% normal saline bolus was administered, and CWI was initiated. A total of 22 minutes elapsed from EMS arrival to CWI initiation. En route to the hospital, the ambulance stopped to obtain additional ice for further cooling. Before ED arrival, a seizure was noted, and 10 mg IV midazolam was administered. The patient’s initial ED rectal temperature 23 minutes after CWI initiation was 39.3 °C (102.2 °F), with a GCS score of 6. Lactic acid was elevated at 4.2 mm/dL, and AST/ALT were mildly elevated to 90 U/L and 53 U/L, respectively. Creatinine level was normal. The patient was intubated in the ED, admitted to the ICU, and had a LOS of 36 days and six hours before being discharged home.

## DISCUSSION

In this case series, prehospital CWI was feasible and effective. Cold-water immersion has been shown to be the prehospital treatment of choice for exertional heat stroke in both military and athletic environments.^5^–^7^ However, few data exist regarding prehospital treatment of undifferentiated heat stroke in a less controlled, more variable emergency dispatch ground-based response setting.

These patients were critically ill with markedly deranged hemodynamics, respiratory status, and altered mental status with associated seizure activity. All patients received 0.9% normal saline fluid boluses, and four required vasopressor support with push-dose epinephrine. However, no patient required long-term vasopressor support via a continuous infusion (which was available under the EMS protocols in effect during the study period). Although hypotension occurred in several of these patients after CWI, its significance to the potential cold shock response remains unclear.^28^,^29^ Since hypotension is an expected effect of rapid cooling, vasopressor preparation and readiness are crucial when initiating CWI.

Three patients were also noted to have reported seizure activity. One patient (case #4) was noted to be seizing prior to CWI initiation, while two other patients (case #5 and #6) developed seizure activity post-CWI. All received appropriate prehospital administration of parenteral midazolam with EMS documentation of seizure activity cessation. Persistent nonconvulsive seizure activity is a possibility, as electroencephalography was not available in the field or ED. Because seizure activity is a known complication of heat stroke,^3^,^4^,^19^ benzodiazepine treatment should be available and prepared when encountering heat stroke and initiating CWI. Two patients required intubation prior to ED arrival, and two additional patients were intubated in the ED upon hospital arrival. The critical nature of heat stroke was further demonstrated by seven of nine patients also requiring ICU admission.

No cases of significant hyperkalemia were noted; however, unsurprisingly, over half (5/9) had lactic acidosis and AKI on ED laboratory investigation. Rhabdomyolysis and significant AST/ALT elevation were both uncommon but were noted in a patient experiencing exertional heat stroke (Case #5). While our small sample size limits detailed analysis, heat stroke should be recognized as a time-sensitive emergency with a high likelihood of hemodynamic instability, seizure, respiratory compromise, and ICU admission. Emergency medical services and ED protocols should be prepared and proactive in anticipating these events.

Logistical challenges arose in initiating prehospital CWI. We did not stipulate where the paramedics should obtain ice and left the decision to their discretion. In some cases, our fire department first-responder partners had water and ice available, whereas in others, paramedics obtained supplies from the patients’ homes and local businesses. Ambulance space is limited, making it difficult for EMS crews to carry ice. Water is readily available in most places; however, ice is more difficult to obtain. In two cases, because no ice was available initially, the patient was immersed in water until the ambulance stopped at a business where bags of ice were obtained and added to the water to improve patient cooling. Future studies should explore methods for carrying and storing ice more efficiently on ambulances, with guidance on the minimum amounts of ice and water required to initiate CWI.

Some aspects of prehospital CWI, however, went smoothly. Monitoring the patient’s vital signs during immersion was successfully accomplished, because the monitoring cables are waterproof. Patients were attached to monitoring before being submerged. While the patient was submerged in water, the wires were left in place for continuous monitoring, and cable connections were kept dry. In prior work, this has been shown to be possible using a continuous rectal monitoring probe.^26^ As in earlier studies, a waterproof immersion bag was used and proved effective.^2^,^5^,^7^,^15^,^17^,^24^,^25^ These bags are relatively inexpensive, easily compactible, and easily stocked on each EMS apparatus.

Ultimately, we feel that CWI is feasible for undifferentiated heat stroke patients presenting in the ground-based, emergency dispatch prehospital setting and should be considered for all EMS protocols. The introduction is uncomplicated and inexpensive, involving only the purchase of waterproof immersion bags and initial training. Despite the apparent benefits and relative ease of implementing prehospital CWI, evidence indicates that EMS protocols remain significantly lacking.^30^ Continuing educational and awareness efforts for EMS medical directors and clinicians are likely warranted.

## LIMITATIONS

Generalizability is limited by the small number of patients treated, the retrospective nature of the data, and the absence of a comparator group in this case series. Due to the pragmatic nature of the immersion protocol, which is intended for use in nonmilitary or athletic event situations, the amount of ice and the timing of ice placement in the water baths were variable. While we did have some laboratory, admission, and mortality data from our hospital partners, additional details, including CWI immersion time, cooling reduction rate, and functional status at discharge, would be desirable in future studies.

## CONCLUSION

In this case series, rapid initiation and maintenance of prehospital CWI for undifferentiated heat stroke were both feasible and effective when initiated by paramedics. These patients were critically ill, with seven of the nine requiring ICU admission; however, there were no reported deaths, and all patients were discharged home. Larger prospective studies are needed to further investigate this treatment.

## Figures and Tables

**Figure.**
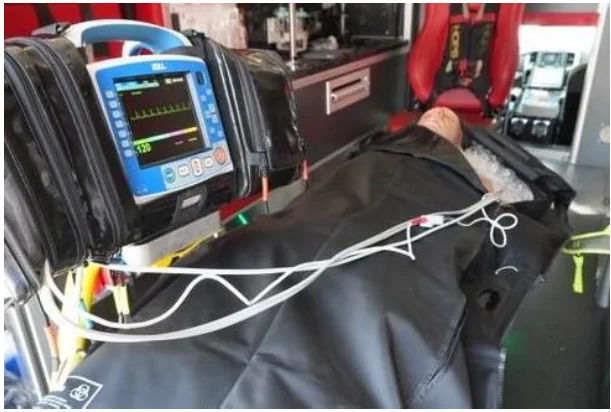
Cold-water immersion protocol. *MD*, medical director; *F*, Fahrenheit.

**Image:**
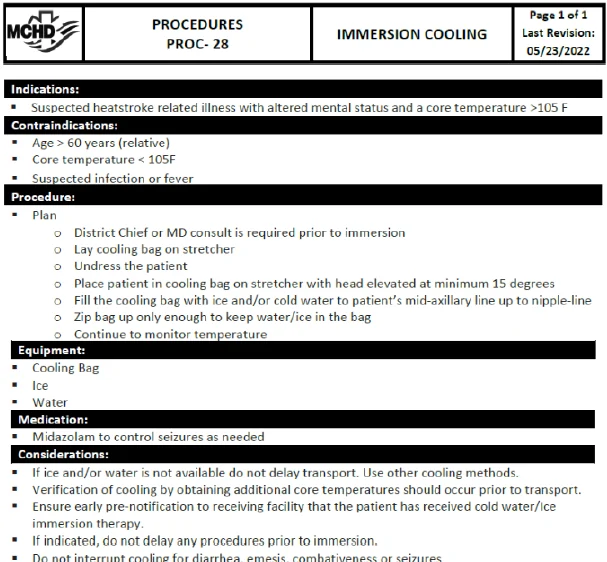
Cold water immersion example.

**Table 1:** Patient data—prehospital**—**used in a case series evaluating the feasibility and effectiveness of prehospital cold-water immersion initiated before/during emergency medical services transport.

Case	Age	Sex	Initial EMS GCS	Initial EMS Pulse (BPM)	Initial EMS BP (mmHg)	Initial EMS Temp (°C)	Final EMS GCS	Final EMS Pulse (BPM)	Final EMS BP (mmHg)	Final EMS Temp (°C)	EMS Scene Time (min)	EMS Transport Time (min)	Time from Immersion to last rectal temp (min)	Time from Immersion to ED arrival time (min)	EMS Intubation (Y/N)
1	67	M	3	160	109/73	41.8	3T	139	154/89	39.6	47	18	48	48	Y
2	72	M	4	124	130/68	41.3	3T	146	193/79	NA	48	24	NA	41	Y
3	78	M	3	132	136/61	40.9	13	113	126/53	39.3	28	10	22	22	N
4	60	F	3	175	85/52	40.9	9	160	92/50	39.7	21	15	17	20	N
5	49	M	7	179	179/71	42.7	7	155	143/83	40.0	16	29	20	20	N
6	51	M	10	111	108/69	40.6	10	98	126/71	39.4	27	13	11	14	N
7	60	M	9	86	150/90	40.7	14	119	109/67	NA	29	31	NA	36	N
8	70	M	12	127	73/48	41.3	15	104	134/73	NA	20	25	NA	40	N
9	50	F	6	122	66/47	42.1	6	110	119/57	39.0	30	20	23	23	N

*BP*,blood pressure; *BPM*, beats per minute; *°C*, degrees celcius; *EMS*, emergency medical services; *F*, female; *GCS*, Glasgow Coma Scale; *M*, male; *mm Hg*, millimeters of mercury; *min*, minute; *N*, No; *NA*, not available; *Y*, yes; *3T*, GCS of 3 and Intubated.

**Table 2:** Patient data—hospital—used in a case series evaluating the feasibility and effectiveness of prehospital cold-water immersion initiated before/during emergency medical services transport.

Case	Admission	ICU	Death	LOS	ED HR (BPM)	ED BP (mmHg)	ED GCS	ED Temp (°C)	ED Lactic Acid (mmol/L)	Initial Potassium (mEq/dL)	Initial AST/ALT (U/L)	Initial Creatinine (mg/dL)	CK (IU/L)	ED Intubation (Y/N)
1	Y	Y	N	7D16H	160	154/89	3T	41.8	3	3.7	50/40	1.6	163	N/A
2	Y	Y	N	9D18H	101	134/62	3T	41.3	5.9	2.2	256/258	8.8	88	N/A
3	Y	N	N	3D20H	113	126/53	14	39.3	1.2	3.8	21/51	4.3	197	N
4	Y	Y	N	5D	170	85/52	14	40.9	2.5	4.5	15/less than 7	1.9	77	N
5	Y	Y	N	7D5H	170	179/71	10	40.0	1.4	3.0	339/245	1.8	10750	Y
6	Y	Y	N	17D19H	98	126/71	14	40.7	1.7	3.1	30/27	1.1	72	N
7	Y	N	N	19H	119	109/67	14	37.3	1.7	3	23/9	0.9	121	N
8	Y	Y	N	14D20H	104	122/76	15	36.4	5.9	4.7	52/92	3.9	159	N
9	Y	Y	N	36D6H	109	72/43	3	39.3	4.2	2.7	90/53	1.1	154	Y

*ALT*, alanine aminotransferase; *AST*, aspartate aminotransferase; *BP*, blood pressure; *BPM*, beats per minute; *CK*, creatine kinase; *°C*, degrees celcius; *ED*, emergency department; *D*, day; *GCS*, Glasgow Coma Scale; *H*, hour; *HR*, heart Rater *ICU*, intensive care unit; *LOS*, length of stay; *min*, minute; *N/A*, not applicable or not available; *N*, no; *Y*, yes; *3T*, GCS of 3 and Intubated.
